# Long-term *ex vivo* maintenance of testis tissues producing fertile sperm in a microfluidic device

**DOI:** 10.1038/srep21472

**Published:** 2016-02-19

**Authors:** Mitsuru Komeya, Hiroshi Kimura, Hiroko Nakamura, Tetsuhiro Yokonishi, Takuya Sato, Kazuaki Kojima, Kazuaki Hayashi, Kumiko Katagiri, Hiroyuki Yamanaka, Hiroyuki Sanjo, Masahiro Yao, Satoshi Kamimura, Kimiko Inoue, Narumi Ogonuki, Atsuo Ogura, Teruo Fujii, Takehiko Ogawa

**Affiliations:** 1 Laboratory of Proteomics, Institute of Molecular Medicine and Life Science, Yokohama City University Association of Medical Science, Yokohama, Kanagawa 236-0004, Japan; 2 Department of Urology, Yokohama City University Graduate School of Medicine, Yokohama, Kanagawa 236-0004, Japan; 3 Department of Mechanical Engineering, Tokai University, Hiratsuka, Kanagawa 259-1292, Japan; 4 RIKEN, Bioresource Center, Tsukuba, Ibaraki 305-0074, Japan; 5 Institute of Industrial Science, University of Tokyo, Komaba, Meguro-ku, Tokyo 153-8505, Japan

## Abstract

In contrast to cell cultures, particularly to cell lines, tissues or organs removed from the body cannot be maintained for long in any culture conditions. Although it is apparent that *in vivo* regional homeostasis is facilitated by the microvascular system, mimicking such a system *ex vivo* is difficult and has not been proved effective. Using the culture system of mouse spermatogenesis, we addressed this issue and devised a simple microfluidic device in which a porous membrane separates a tissue from the flowing medium, conceptually imitating the *in vivo* relationship between the microvascular flow and surrounding tissue. Testis tissues cultured in this device successfully maintained spermatogenesis for 6 months. The produced sperm were functional to generate healthy offspring with micro-insemination. In addition, the tissue kept producing testosterone and responded to stimulation by luteinizing hormone. These data suggest that the microfluidic device successfully created *in vivo*-like conditions, in which testis tissue maintained its physiologic functions and homeostasis. The present model of the device, therefore, would provide a valuable foundation of future improvement of culture conditions for various tissues and organs, and revolutionize the organ culture method as a whole.

The history of *in vitro* spermatogenesis dates back to about a century ago. It parallels the history of organ culture methods, because *in vitro* spermatogenesis was performed mostly using the organ culture method until around the 1970’s^1^. Although there are several ways to culture a tissue fragment or small organ, the golden standard has long been the so-called interphase method in which samples are placed at the interphase between the culture medium and a gas layer^2^. We adopted this classical organ culture method for culturing mouse testis tissues, and succeeded in inducing complete spermatogenesis, from spermatogonial stem cells up to fertility-competent sperm formation^3^,^4^,^5^. Nonetheless, the overall efficiency and duration of the spermatogenesis were far from comparable to those observed *in vivo*. In the body, blood capillaries surrounding a unit of tissue supply nutrients and oxygen and remove waste products efficiently and continuously, promoting the homeostasis of the regional tissue. The interphase method lacking such a microcirculatory system, therefore, cannot provide conditions that are comparable with those *in vivo*.

To overcome this, researchers introduced circulatory mechanisms to their culture systems. In particular, Rose and colleagues developed a culture system, named the circumfusion system in which circulating medium flows at a high speed on cultured tissue covered with a cellophane membrane^6^,^7^. Various organs cultured in this system were reported to maintain their morphological features for an extended period of several months. Unfortunately, however, the method was not fully evaluated in terms of tissue functions at that time, and it has been rarely examined until the present. Recently, on the other hand, microfluidics (MF) has been applied into cell culture experiments^8^,^9^,^10^,^11^. A microfabricated chip, made of polydimethylsiloxane (PDMS)^12^ and having a set of micro-channels etched or molded into it, can serve as a culture vessel for cells and can easily accommodate medium flow or circulation^13^. Tissue pieces, such as brain or liver slices, have also been cultured in MF devices to maintain their function more favorably than conventional culture methods^14^,^15^. Now, it has become possible with MF technologies to test whether this idea, incorporating circulatory systems into a culture system to induce and maintain tissue functions for an extended period of time, is really effective or not. Recently, in fact, researchers succeeded in extending the culture period of tissues or cell aggregates in MF devices for as long as several weeks^16^,^17^, and the importance of long-term culture is beginning to be recognized^18^.Through our studies using MF technology, we have developed and produced devices for culturing embryos, intestinal epithelial cells, liver cells, and iPS cells^19^,^20^,^21^,^22^. One of the features of these MF devices was the introduction of porous membranes to separate chambers or channels. Such membrane-laden devices came to be used effectively for cell cultures by other researchers^23^,^24^,^25^. In the present study, based on such experience, we have devised a MF device incorporating a porous membrane which separates the cultured tissue from a slowly flowing medium. We found that mouse testis tissue fragments loaded in this device successfully maintained spermatogenesis and testosterone production efficiently for extended periods of time.

## Results

### Limitation of classical interphase method

In this project to maintain spermatogenesis *in vitro* for extended periods of time, re-evaluation of the interphase method, or agarose gel (AG) method^3^,^5^, was initially performed. Using *Acr-Gfp* transgenic mice, which express GFP in spermatogenic cells from the mid-meiotic phase onward (Supplementary Fig. S1), the extent of spermatogenic progression was evaluated by the GFP-grade, which classifies the spread of GFP expression in each tissue into 5 grades (Supplementary Fig. S2A). In the experiment culturing 73 tissues in total, the average GFP-grade peaked at 4-week with the value of over grade 4 and declined gradually to around grade 1 after 18-week (Supplementary Fig. S3A). At 24 weeks, GFP expression was observed in 9 tissues (12.3%) and spermatids were confirmed in 4 (5.5%). These long-lasting GFP expression and foci of spermatogenesis histologically confirmed were unexpected and rather surprising. They were, however, sporadic and limited in a small region of the tissue. In addition, throughout our experiments, it was not possible to identify key factors that can induce such long-term spermatogenesis. Thus, the phenomenon remained infrequent and uncontrollable. One interesting finding that was different on comparing early GFP expression and long-lasting expression was their location in the tissue. Regular GFP expression, the focus of spermatogenesis, starts at the periphery of each tissue piece (Supplementary Fig. S3B). This topological feature, however, did not apply to the long-lasting GFP expression foci. They appeared to locate inside of the tissues (Supplementary Fig. S3C). Histological examination also showed that seminiferous tubules harboring spermatogenesis were observed not at the edge but submarginal inner region of the tissues (Supplementary Fig. S3C). This observation suggested that the direct exposure of seminiferous tubules to air and medium was disadvantageous when attempting to maintain spermatogenesis for a long period.

### Molecular diffusion through testis tissue

The distance that nutritional molecules can diffuse through the testis tissue was an important issue on designing the MF device. This molecular diffusion distance was measured using a prototype MF device made of PDMS, named the slit device (Supplementary Fig. S4). In this device, the sample tissue was placed in a tissue chamber which was partitioned from medium flow channel by aligned pillars, slits. Regions in testis tissue that remained viable were within a distance of 300  ~  400 μm from the pillar slits, indicating that nutritional diffusion could reach such a distance (Supplementary Fig. S5). The experiment with this device not only gave us data on the diffusion distance, but also revealed that the separation of the sample tissue from flowing medium might be better for testis tissues in a microfluidic device.

### Design of the MF device

**Figure 1:**
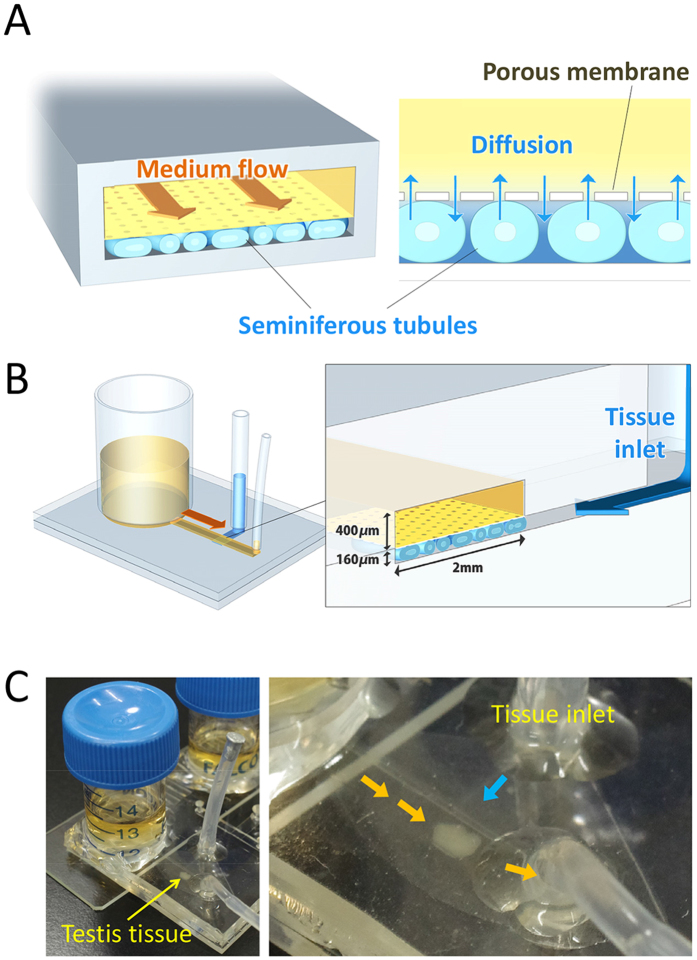
The microfluidic device. (**A**) The concept of the device was to separate medium flow and the tissue by a porous membrane. (**B**) Schematic 3-D image of the device. (**C**) A picture of the device with testis tissue loaded in the chamber.

Based on the information above, the MF device was designed (Fig. 1A). The device uses a thin porous membrane, 10 μm-pore polycarbonate, to separate sample tissues and the flowing medium. The medium flow channel (2 mm in width and 400 μm in height) was expected to serve like a capillary vessel to deliver a continuous supply of fresh culture medium (Fig. 1B,C). The sample tissue chamber was 2 mm × 3 mm × 160 μm (W × L × D) which allows the sufficient diffusion of molecules through the membrane and in the tissue to its base. As the diameter of seminiferous tubules grows from 50 ~ 70 μm at birth to around 200 μm in the adult stage, a chamber depth of 160 μm was considered to allow nearly full tubule development in the chamber. This chamber space accommodated a single testis of neonates and its growth was observed based on the decrease in the free space as it grew. When a testis of older mice was used and filled the space at loading, growth was not clearly noted but it extended toward the space of the inlet channel.

### Spermatogenesis in the MF device

**Figure 2:**
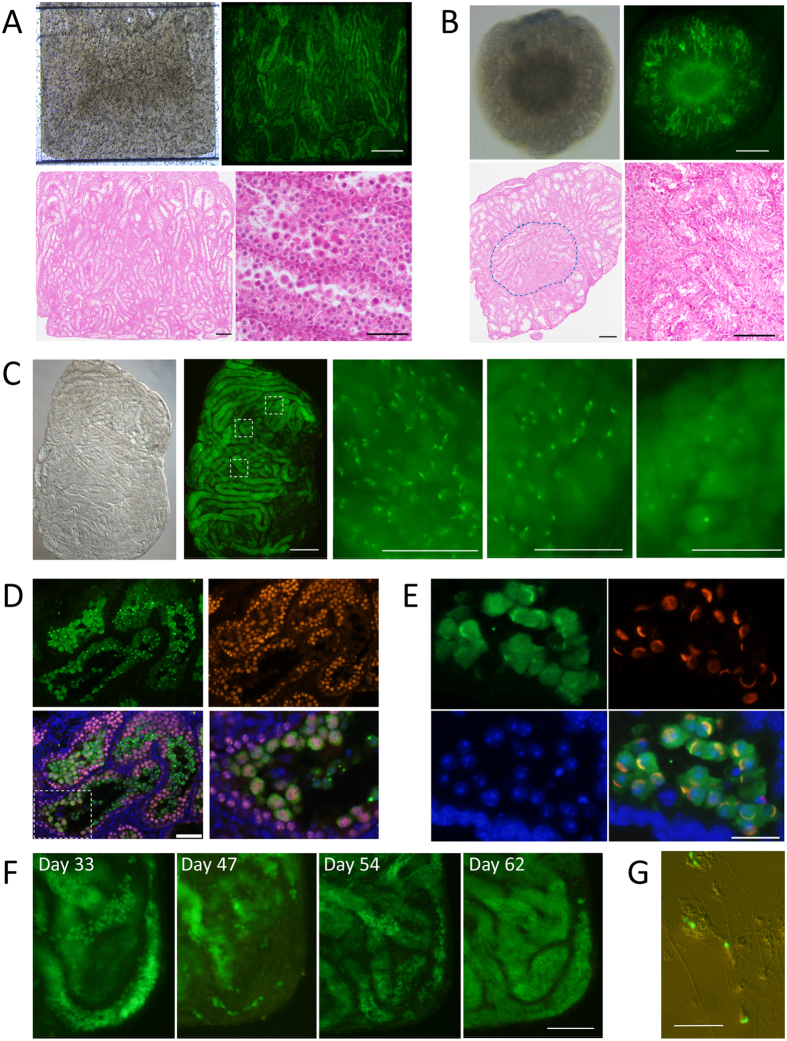
Progression of spermatogenesis in the microfluidic device. (**A**) Testis tissue was spread flat in the tissue chamber. GFP expression was observed in most tubules, observed on culture day 42. Histological examination confirmed that most seminiferous tubules contained germ cells in the meiotic phase (HE). (**B**) Tissues cultured with the AG method showed GFP expression in a donut-like fashion, with the central region degenerated. HE staining histology demonstrated such changes. The circled area is magnified in the right lower panel showing the degenerative tubules. (**C**) Testis tissue of 4.5 dpp mice cultured for 38 days in the device. Central areas of the 3 dashed rectangles are enlarged in the right panels, showing that each portion is in a different stage of spermatogenesis. (**D**) Immunohistochemical examination with antibodies to GFP (green) and SCP3 (red), counterstained with Hoechst (blue). The bottom left panel is the merged image. The dashed rectangular area is enlarged in the bottom right panel. The GFP(−)SCP3(+) cells were observed surrounding the GFP(+)SCP3(+) cells inside, along with inner-most GFP(+)SCP3(−) cells, confirming that *Acr-Gfp* expression starts in the mid-pachytene stage of meiosis and protein remains in round spermatids. (**E**) Lectin PNA staining (red) demonstrated that GFP-dots and -caps corresponded to the acrosome structure. (**F**) A single spot, time-course observation revealed that GPF-positive cells repeated emergence and disappearance. (**G**) Sperm with GFP-acrosomes and flagella were found in the suspension produced by tissue dissociation. Scale bars: 500 μm (**A**,**B** upper, **C** left), 200 μm (**A**,**B** lower, **F**), 100 μm (**D**), 50 μm (**C** right), 20 μm (**E**,**G**).

The initial experiment was performed using testis tissues of a 0.5-day-old *Acr-Gfp* mouse. At this age, the seminiferous tubules contain only gonocytes as germ cells, precursors of spermatogonia (Supplementary Fig. S6) In the chamber, each seminiferous tubule was clearly visible with a stereomicroscope, and GFP expression was confirmed from culture day 21 up to day 42, when it was processed for histological examination (Fig. 2A). As a consequence of the tissues being spread flat in the chamber and exposed evenly to the flowing medium, spermatogenesis occurred in most seminiferous tubules. In this sample, the percentage of tubules expressing GFP was over 70%, corresponding to GFP-grade 5 (Supplementary Fig. S2B). In fact, histological examination confirmed that 89.4% (185/207) of the seminiferous tubules contained germ cells in the meiotic phase, identified by their location in the tubule and characteristic chromatin appearance (Fig. 2A). This was a highly efficient and widespread induction of spermatogenesis under *ex vivo* conditions, even though it was not comparable with the mature testis *in vivo*, which is almost 100%, and each seminiferous tubule contains 4 layers of germ cells including those in the meiotic phase in the second layer (Supplementary Fig. S6). In the case of the AG method, on the other hand, such widespread induction of spermatogenesis did not occur because seminiferous tubules on the agarose gel coalesced to form a dome-like structure even when they were spread flat initially. In many cases, therefore, the central region received insufficient nutrients and oxygen, resulting in degenerative or necrotic changes of the tissue (Fig. 2B).

The MF device was able to offer high visibility, being compatible with the inverted microscope. The objective lens of a microscope can get close to the tissue from the bottom of the lower PDMS layer. By observing the tissue in a microfluidic device closely, it was recognized that different portions of the tissue showed different stages of spermatogenesis (Fig. 2C). These GFP-expressing cells corresponded with the mid- and late-meiotic cells, confirmed by immunohistochemistry with Synaptonemal complex protein 3 (SCP3) antibody, a specific marker of pachytene meiosis (Fig. 2D). The GFP-positive acrosomes were stained with peanut agglutinin (PNA) lectin, an acrosome marker, confirming the fidelity of *Acr-Gfp* as a marker of spermatogenic progression (Fig. 2E). When a particular portion of the tissue was observed over time, the emergence of GFP-positive acrosomes indicated the progression of spermatogenesis (Supplementary Fig. S7). It was also observed that GFP-positive cells in the tubule on day 33 disappeared on day 47. The same tubule grew new GFP-positive cells on day 54, reaching a peak on day 62 (Fig. 2F). In addition, when the tissue was removed from the device and mechanically dissociated, sperm with GFP-positive acrosomes and flagella were collected (Fig. 2G). These observations demonstrated that the spermatogenesis, up to sperm formation, occurred continually throughout the culture period in the device.

### Long-term culture

**Figure 3:**
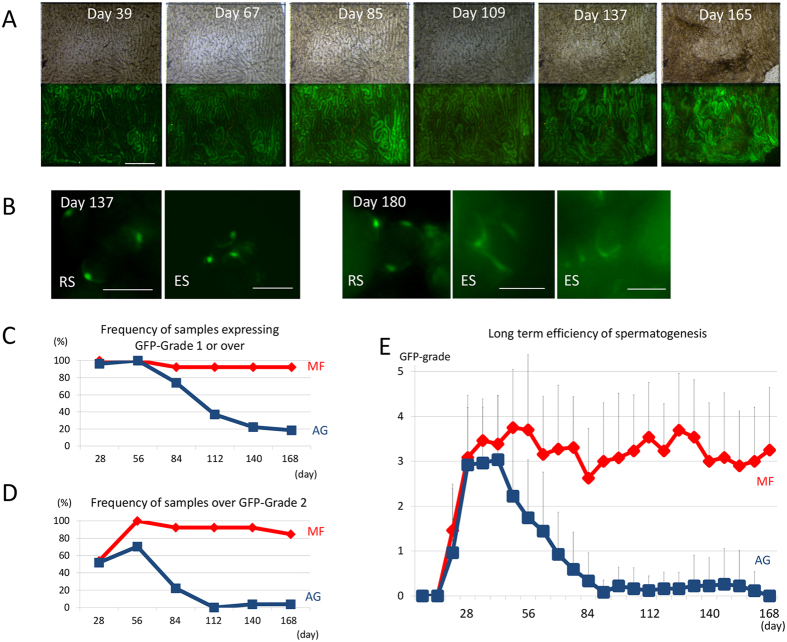
Long-term culture experiments. (**A**) A testis tissue fragment, from *Acr-Gfp* neonates, at 0.5 dpp, was cultured in MF, and GFP expression lasted throughout the period. (**B**) In the same tissue, by magnification, GFP-positive acrosomes were observed intermittently even in the late culture period. (**C**,**D**) MF and AG were compared based on the frequency of tissue samples expressing GFP grade 1 or above (**C**) and over 2 (**D**). (**E**) The average GFP-grade transition was compared in MF and AG. Scale bars: 500 μm (**A**), 10 μm (**B**).

Next, the durability of the spermatogenesis in the MF device was tested by continuing each experiment for longer periods. In a single experiment, the GFP expression was maintained at a high level for 180 days (Fig. 3A). Haploid cells were observed intermittently until the end of culture, such as on days 137 and 180 (Fig. 3B). To confirm this result of long-term culture, the GFP grade of tissues in the MF device was recorded for 180 days and compared with that of the AG method. Although GFP grades with MF and AG methods were not equivalent, it was fair and reliable to assess the expansion of spermatogenesis in the MF device (Supplementary Fig. S2A, B). Among the 13 tissue samples loaded in the MF device, 12 maintained GFP expression for up to 24 weeks, while only 5 out of 27 maintained GFP with the AG method (Fig. 3C). When only GFP-grade 3 or above was counted, the difference in the maintenance ratio was more marked: 11 out of 13 with MF and 1 out of 27 with AG method (Fig. 3D). The average GFP grade with the MF method was maintained at around 3, while that with the AG method dropped down below 1 in 10 weeks and was close to 0 after 13 weeks (Fig. 3E).

### Offspring production

**Figure 4:**
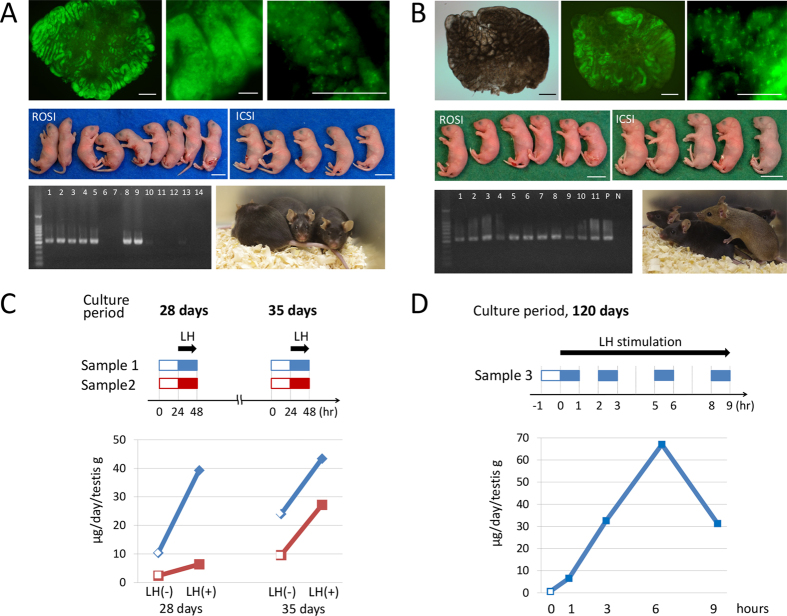
Micro-insemination experiments and testosterone production. (**A**) Testis tissue cultured for 41 days included many spermatids (upper panels), which was dissected for harvesting haploid cells. ROSI and ICSI procedures produced healthy offspring (middle panels). Genomic PCR demonstrated that 7 out of 14 offspring carried the GFP gene, revealing their origin to be the transgenic sire, heterozygous for *Acr-Gfp* (lower left panel). They normally matured and were observed on 346 dpp (lower right panel). (**B**) Testis tissue cultured for 185 days was used for microinsemination (upper panels). Both ROSI and ICSI produced healthy offspring (middle panels). Genomic PCR demonstrated that all 11 offspring carried the GFP gene, revealing their origin to be the transgenic sire, homozygous for *Acr-Gfp*. Lanes P and N were loaded with a positive-control sample (genomic DNA of *Acr-Gfp* mouse) and a negative-control sample (genomic DNA of ICR mouse), respectively (lower left panel). The pups normally matured and were observed on 66 dpp (lower right panel). (**C**) Testosterone production by two cultured testis tissues, sample 1 & 2, was measured at 4 and 5 weeks by collecting medium flowing out of the device for 24 hours each. LH was added to the culture medium on the next day and medium samples were collected in the same way for testosterone measurement. (**D**) Testis tissue cultured for 120 days produced testosterone and responded to LH at 1 hour and onward. Scale bar: 1 cm (**A**,**B** middle panels), 500 μm (**A** upper left panel, **B** upper left & middle panels), 100 μm (**A** upper middle & right panels), 50 μm (**B** upper right panel).

Then, fertility-competence of the haploid cells produced in the MF device was evaluated by performing micro-insemination experiments. Firstly, haploid cells were harvested from 2 tissue samples which were cultured for 41 days. Round spermatid injection (ROSI) and intracytoplasmic sperm injection (ICSI) procedures were successfully performed to produce 9 and 5 offspring, respectively. They all grew normally (Fig. 4A, Supplementary Table S1). Next, haploid cells were collected from testis tissue cultured for 185 days which contained numerous spermatids and sperm. Micro-insemination procedures produced 6 and 5 healthy offspring with ROSI and ICSI, respectively. They grew healthily and matured into adults (Fig. 4B, Supplementary Table S2). These results demonstrated that spermatogenesis in microfluidic devices can produce haploid cells that are competent male gametes.

### Testosterone production

Lastly, the hormone-producing ability was tested of the tissue cultured in the MF device. By electro-chemiluminescence immunoassay (ECLIA), testosterone was measured in the “used” culture medium which passed through the microchannel above the tissue. Four samples obtained from a whole day’s medium flow were collected from 2 testis tissues in culture weeks 4 and 5. All of them showed testosterone production of 2.4 ~ 24.0 (μg/day/g testis) which is comparable to the production by the testis in the body^26^,^27^. In addition, the production increased by 1.8 to 3.8-fold when luteinizing hormone (LH) was added to the culture medium (Fig. 4C). A tissue cultured for 120 days was also examined. In this case, the medium was collected for 1 hour at 0, 1, 3, 6, and 9 hours after LH stimulation. Liquid chromatography-tandem mass spectrometry (LC-MS/MS) was used which is more sensitive and specific for steroid measurement. The basal production rate, at 0 hours, was measured and calculated to be 0.63 (μg/day/g testis). After LH addition, it rose at 1 hour and onward by 10.3 to 106-fold (Fig. 4D). These data, although preliminary and based on a single sample in a single experiment, showed that the testis tissue maintained its ability to produce testosterone as well for 4 months, which is much longer than previously achieved.

## Discussion

Long-term culture, or maintaining the architectural as well as functional integrity of an organ *ex vivo* for an extended period of time, has long been a challenge for researchers conducting culture experiments. In the past, it was reported that hamster lung and human skin tissues cultured for up to 6 weeks were viable for that period^28^,^29^. As for testicular tissue, it was reported that rat testis tissue fragments could be maintained for 8 weeks while preserving the basic architecture of the seminiferous tubules along with their functional potential, which was demonstrated by the reappearance of spermatogenesis after grafting to host testis of immune-compatible rats^1^. This does not mean, however, that spermatogenesis was maintained during the culture period; primitive spermatogonia alone as germ cells remained in the tissue. Taking these and other time-honored reports together, it could be said that maintaining viable tissue fragments in culture for an extended period, for weeks and months, should be possible, but expressing their functions for such periods is extremely difficult, leaving this task rarely addressed in recent decades.

In the present study, we demonstrated that neonatal mouse testis tissue fragments loaded in our simple microfluidic device showed a more efficient induction of spermatogenesis than with the conventional interphase method, and this was consistently maintained for a long period of 6 months. Sperm produced in testis tissue cultured for 6 months was competent to produce normal healthy offspring. In addition, the tissue also maintained its endocrine function. To our best knowledge, this is the first demonstration of such a long-term maintenance of a tissue function *ex vivo*, regardless of the tissue origin.

We initially hypothesized that tissues in a microfluidic device can benefit from the efficient exchange of molecules by flowing culture medium through the tissue surface^9^,^10^,^11^. However, our finding obtained by long-term culture with the AG method where spermatogenesis was maintained in the submarginal inner but not at the most peripheral regions of the tissue, was rather paradoxical and informative. With this in mind, we reason that, in addition to the efficient exchange of molecules between the culture medium and testis tissue, there are at least two explanations of the present results of our long-term culture. Firstly, the sample tissues in the MF received oxygen through PDMS. This condition may reduce the toxicity of oxygen compared to direct exposure^12^, and would have contributed to the longevity of the tissue. Secondly, the flowing culture medium in the channel does not pass through the membrane into the tissue chamber. The exchange between the flowing culture medium and fluid in the tissue chamber occurs principally by molecular diffusion. This relationship seems basically similar to that between blood plasma and tissue interstitial fluid in the body; thus, this would be another key factor that contributed to the present results. Interstitial fluid is made up of two components: exudate from capillary plasma and secretions from tissue. In addition, the fluid is under constant drainage from the capillary and lymph vessels along with selective absorption by the tissue. This chemical milieu of interstitial fluid, produced by the tissue itself in part, should play a pivotal role in maintaining the tissue function. We suggest that the tissue chamber in the MF also helped to produce the specific chemical milieu of the testis tissue, as the chamber was half-separated from the flowing medium by the porous membrane, which would have maintained secreted molecules inside the chamber for a longer period than if open to diffuse away freely. A similar condition may have been incidentally produced at the submarginal regions of the tissue in the AG experiment. This discussion, however, may contradict the previous argument of “efficient molecular exchange” between the medium and tissue. It is important, therefore, to find a good balance between these two requisites. In reality, such a balance could be searched for and achieved more easily with the MF system than ordinary culture system. Thus, we suggest that our MF device succeeded in better fulfilling these requirements than the AG method by achieving a good balance between them.

We successfully improved the culture conditions for testis tissues in the present study. However, they could be optimized further by more precisely setting parameters, such as the medium flow speed, pore size and porosity of the membrane, tissue chamber size and especially its height, and thickness of PDMS walls. We believe that such optimization of the culture conditions along with improvement of the culture medium would revolutionize the organ culture method in general, leading to the realization of “organs-on-chips” technology.

## Materials and Methods

### Design of microfluidic device

The microfluidic device consists of two PDMS (polydimethyl- siloxane, Silpot 184, Dow Corning) layers, upper and lower, and a thin porous polycarbonate membrane (Pore size: 10 μm, porosity: 5–20%, Isopore membrane filter, Millipore). The upper PDMS layer has microchannels for medium flow, which are 2 mm wide, 400 μm high, and run for 2 cm from a tank to an outlet. The lower layer, on the other hand, has a tissue-loading channel and tissue chamber, which is 3 mm wide, 160 μm deep, and 2 mm long. Between the flow channel and tissue chamber, the porous membrane is set to separate them. The thickness of the PDMS layers is between 1 and 1.5 mm.

### Device fabrication

The microfluidic device was produced by conventional photolithography and soft lithography techniques^30^,^31^,^32^. First, a mold master made of a negative–type photoresist (SU-8 2100; MicroChem Co.) was prepared, which serves as the mold for the production of PDMS layers, upper and lower. The SU-8 was first poured on a 4-inch wafer and spin-coated over it to achieve the target thickness, such as 160 and 400 μm, evenly over the wafer. They were then pre-baked, and patterned by UV light exposure through a photomask for several seconds to carve a pattern of channels, followed by post-baking. The photomask was designed with CAD software (AutoCAD: Autodesk, Inc.) and fabricated with a laser lithography system. The baked mold master was developed by incubation in (1-methoxy-2-propyl) acetate (Merck/code 818532/ Tokyo/Japan) for 10 minutes, followed by rinsing in isopropanol (Kanto Chemical /code 32435-70/Kanto/Japan) for 3 minutes. This SU-8 mold master can be used repeatedly for the replica molding of PDMS layers. For the production of each device, PDMS prepolymer was mixed with a curing reagent (Silpot 184, DowCorning.) at a 10:1 weight ratio and poured over the mold master. It was placed in a vacuum chamber for degassing and then moved to an oven to be heated at 75 °C for 2 hours for curing. After cooling down, solidified PDMS was peeled off the master. Holes were drilled into the upper layer of PDMS for medium inlet and outlet, and tissue inlet. To bond together, bonding surfaces of two layers of PDMS were treated with oxygen plasma activation in a plasma cleaner apparatus (Expanded Plasma Cleaner PDC-32G, Harrick Plasma). The thin porous polycarbonate membrane, which was to be sandwiched between two PDMS layers, was also treated in the plasma cleaner and then dipped in an aminosilane coupling agent to coat it with aminosilane (SILQUEST A-1100 SILANE, Tanac)^32^. Then, PDMS layers and the porous membrane were aligned and bonded together. Silicone tubes for tissue inlet and medium outlet, and a medium reservoir tank, which was the cut half of a polypropylene tube (Falcon™ 15 mL Conical Centrifuge Tubes, Corning), were fixed by PDMS. Devices were sterilized by UV irradiation before use. The outlet tube was connected to a fluorinated ethylene propylene tube (Dupont FEP tubing 1527, IDEX (inner diameter: 0.25 mm)), which was connected to a disposable syringe set in a syringe pump (MFS-SP10X, Microfluidic System Works Inc.).

### Animals

*Acr-Gfp* transgenic mice^33^,^34^ which were provided by RIKEN BRC through the National Bio-Resource Project of MEXT, Japan, were used as a testis tissue source. For mating, males homozygous for *Acr-Gfp* were used as sires, while females were either homozygous, heterozygous, or the wild-type. B6D2F1 female mice (Japan SLC, Inc.) and ICR female mice (CLEA Japan, Inc.) were used for oocyte collection and embryo transfer, respectively. Mice were housed in air-conditioned rooms, 24 ± 1 °C and 55 ± 5%, with a 14 hour-light and 10 hour-dark lighting cycle. The mice were kept in a specific pathogen-free room. Commercially made hard pellets (MF, Oriental Yeast) were fed ad libitum. Drinking water was acidified to pH 2.8–3.0 by HCl. All animal experiments conformed to the Guide for the Care and Use of Laboratory Animals and were approved by the Institutional Committees of Laboratory Animal Experimentation (Animal Research Center of Yokohama City University, Yokohama, Japan, and RIKEN Tsukuba Institute, Japan).

### Culture of testis tissues

Testes of neonatal mice, 0.5–5.5 days postpartum (dpp), were decapsulated. They were used as a whole, and randomly allocated to the MF or AG group for culturing in microfluidic devices or on agarose gels, respectively. As for the MF method, tissue was loaded into the tissue chamber through the tissue inlets. The culture medium was drawn through the outlet by the syringe pump at 0.05 μL/min. Tissues of the AG group were cultured on agarose stands (1.5% w/v) placed in wells of a 12-well culture plate (CELLSTAR® Tissue Culture Plates, Greiner Bio-One)^3^,^4^,^5^. Each gel was loaded with 1–3 testis tissue fragments. The amount of medium was adjusted so that it would come up to half-to-four-fifths of the height of the agarose gel (approximately 0.5 mL/well). Medium change was performed once a week. The culture incubator was supplied with 5% carbon dioxide in air and maintained at 34 °C. The culture medium used for organ culture was α-Minimum essential medium (α-MEM) (Invitrogen: 12000-022), supplemented with 40 mg/mL AlbuMAX (Invitrogen: 10828-028).

### Gross and histological examination

Tissues in culture were observed every 7 days under a stereomicroscope (Leica M 205 FA; Leica, Germany) to evaluate the efficiency of spermatogenesis by the GFP-expression grade. The area showing GFP expression was estimated visually and classified into one of five grades: G0 to G5 (Supplementary Fig. S2), based on our previous GFP-expression grading scale^3^,^4^,^5^. Briefly, G0 to G5 corresponds to 0, ~10, ~30, ~50, ~70, and ~100% of the area of GFP expression, respectively, on observation with stereomicroscopy. An inverted microscope (Olympus IX 73; Olympus, Tokyo, Japan) was used to observe the GFP-expressing germ cells more closely to distinguish steps of spermatids. For histological examination, the specimens were fixed with Bouin’s fixative and embedded in paraffin. One section showing the largest cut surface was made for each specimen and stained with hematoxylin and eosin (HE) or Periodic acid-Schiff (PAS). To search for spermatids and sperm, cultured tissues were mechanically dissociated using needles to release cells into the phosphate-buffered saline (PBS). The cell suspension was observed with a microscope under GFP excitation light.

### Immunohistochemical examination

For immunofluorescence staining, tissues fixed with 4% paraformaldehyde in PBS were cryo-embedded in OCT compound (Sakura Finetechnical) and cut into 7-μm-thick sections. Nuclei were counterstained with Hoechst 33342 dye. Specimens were observed with a microscope (Olympus IX 73; Olympus, Tokyo, Japan). The following were used as primary antibodies: rat anti-GFP antibody (1:1,000, Nakalai Tesque, Inc., Kyoto, Japan), mouse anti-SCP3 antibody (1:200, Abcam) and rabbit anti-peanut agglutinin antibody (1:400, Life technologies). The secondary antibodies used were mouse anti-mouse IgG, goat anti-rabbit IgG, and goat anti-rat IgG, conjugated with Alexa 488 or Alexa 555 (1:200; Life technologies).

### Micro-insemination

The cultured testes tissues were dissected under a stereomicroscope to collect spermatids and spermatozoa. Round spermatid injection (ROSI) and intracytoplasmic sperm injection (ICSI) were performed according to the methods previously reported^35^,^36^. Fertilized oocytes were cultured for 24 h, and two-cell embryos were transferred into the oviducts of pseudopregnant ICR females at 9 weeks old, mated with vasectomized male mice the day before. Live fetuses retrieved on day 19.5 were raised by lactating foster ICR dams.

### PCR analysis

Genomic DNA was extracted from the mouse tail with a DNeasy Tissue kit (Qiagen). The DNA samples (10 ng) were added to a 20-mL reaction mixture containing 0.25 mM of each enhanced green fluorescent protein (EGFP) specific primer and Premix Ex Taq (Takara Bio). EGFP-specific primers were 5′-TACGGCAAGCTGACCCTGAA-3′ and 5′-TGTGATCGCGCTTCTCGTTG-3′. The reaction profile was 30 cycles of denaturation at 95 °C for 60 seconds, annealing at 60 °C for 30 seconds, and extension at 72 °C for 60 seconds.

### Testosterone measurement

The testosterone concentration in sample media was measured by ECLIA (Elecsys® Testosterone II, Roche) or LC-MS/MS (ASKA Pharma Medical). The former assay had a lower limit of sensitivity of 0.025 ng/mL with average intra- and inter-assay variations of 1.3–3.6 and 1.1–3.5%, respectively. The later assay had a lower limit of sensitivity of 1 pg/assay with average intra- and inter-assay variations of 1.4–3.5 and 3.4–5.1%, respectively. The sample media collected in a syringe for 24 hours or 1 hour were subjected to testosterone measurement. For LH stimulation experiments, luteinizing hormone (Sigma: L6420) was added to the culture medium at a final concentration of 1 ng/mL.

## Additional Information

**How to cite this article**: Komeya, M. *et al.* Long-term *ex vivo* maintenance of testis tissues producing fertile sperm in a microfluidic device. *Sci. Rep.* **6**, 21472; doi: 10.1038/srep21472 (2016).

## Supplementary Material

Supplementary Information
