# Supplementary material for: Long-term ex vivo maintenance of testis tissues producing fertile sperm in a microfluidic device
Source: Sci Rep. 2016 Feb 19;6:21472. doi: 10.1038/srep21472 (PMC4759809; doi:10.1038/srep21472)
Supplement: Supplementary Information [file srep21472-s1.pdf]

Long-term ex vivo maintenance of testis tissues producing fertile sperm in a microfluidic device

Mitsuru Komeya[1, 2], Hiroshi Kimura[3], Hiroko Nakamura[3], Tetsuhiro Yokonishi[1,2 †], Takuya Sato[1], Kazuaki Kojima[1], Kazuaki Hayashi[1], Kumiko Katagiri[1], Hiroyuki Yamanaka[1, 2], Hiroyuki Sanjo[1, 2], Masahiro Yao[2], Satoshi Kamimura[4], Kimiko Inoue[4], Narumi Ogonuki[4], Atsuo Ogura[4], Teruo Fujii[5, *] & Takehiko Ogawa[1, 2, *]

1 Laboratory of Proteomics, Institute of Molecular Medicine and Life Science, Yokohama City University Association of Medical Science, Yokohama, Kanagawa 236-0004, Japan.

2 Department of Urology, Yokohama City University Graduate School of Medicine, Yokohama, Kanagawa 236-0004, Japan.

3 Department of Mechanical Engineering, Tokai University, Hiratsuka, Kanagawa 259-1292, Japan.

4 RIKEN, Bioresource Center, Tsukuba, Ibaraki 305-0074, Japan.

5 Institute of Industrial Science, University of Tokyo, Komaba, Meguro-ku, Tokyo 153-8505, Japan.

† Present address: Department of Cell Biology, Duke University, Durham, NC 27710, USA.

* Correspondence should be addressed to T.F. (tfujii@iis.u-tokyo.ac.jp) or T.O. (ogawa@yokohama-cu.ac.jp)

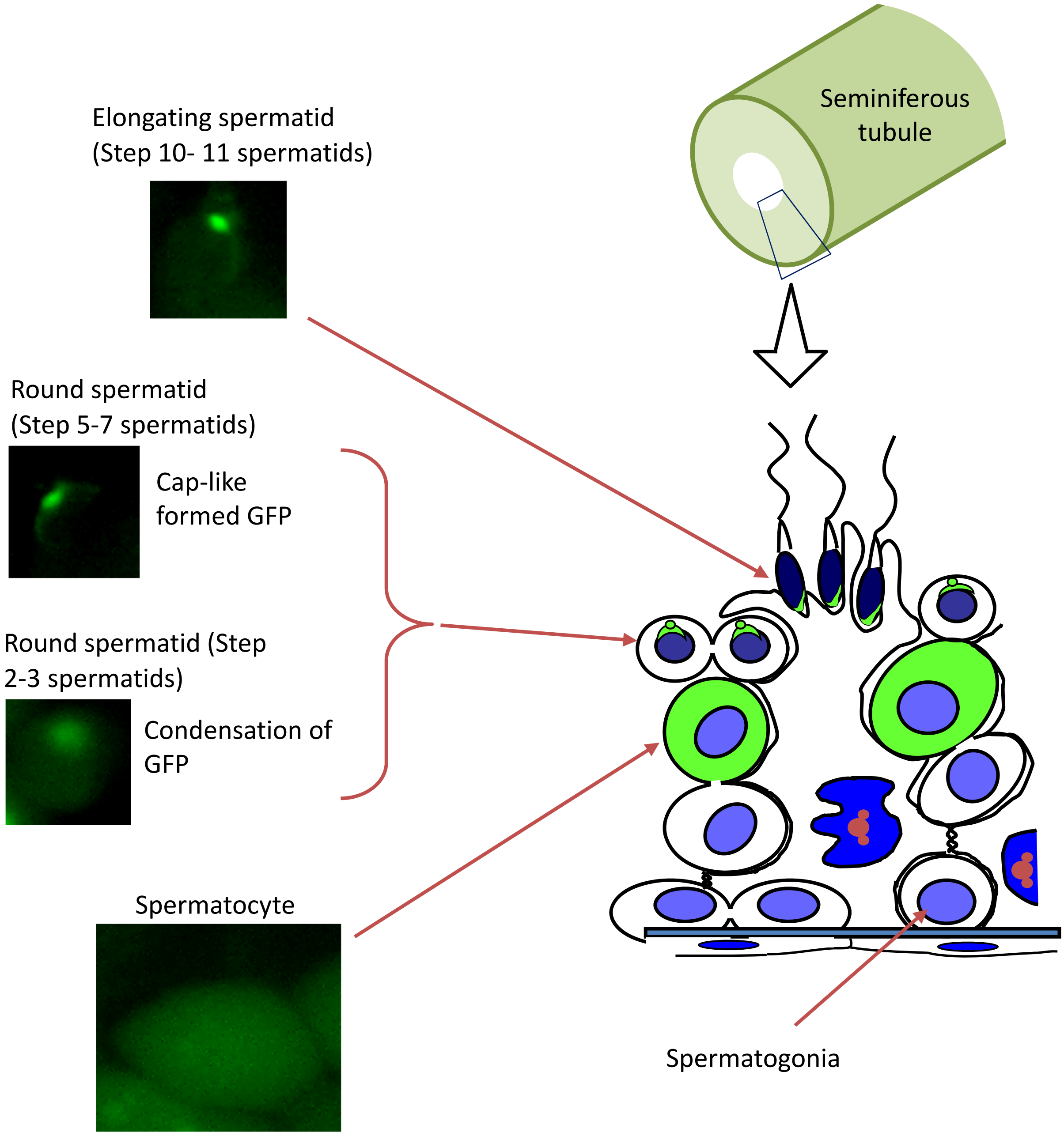

Fig. S1. GFP expression in the *Acr*-GFP mouse testis. Spermatogenic cells of the *Acr*-GFP mouse start expressing GFP in the mid-meiotic phase. The GFP moves and accumulates in the acrosome. As the form of acrosome changed from a dot to cap-like structure, they were observed as GFP-emitting structures.

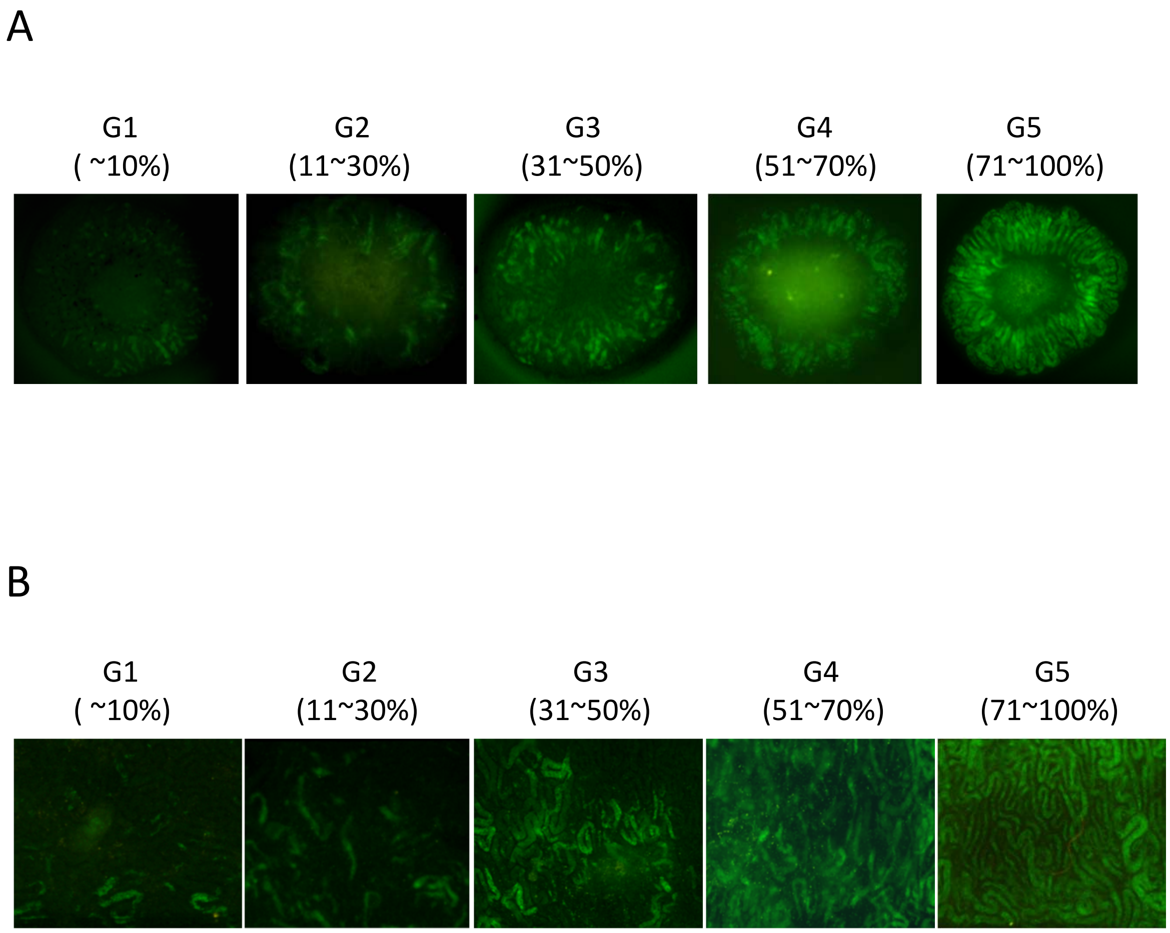

Fig. S2. GFP expression grade for AG (*A*) and MF *(B*) methods. The criterion on judging G1, but not G0, was to observe a single or more stretches of GFP-positive tubules. G1 was assigned when up to 10% of the total tubule area was occupied by GFP-positive tubules. In a similar manner, as the GFP-positive tubules increased from over 10 to 30%, over 30 to 50%, over 50 to 70%, and over 70 to 100%, GFP grades from G1 to G5 were assigned, respectively. In grading AG samples, the central area, which generally undergoes degenerative change, was omitted from the evaluation. While, in the MF, the entire area of the spread tissue was included for judgement.

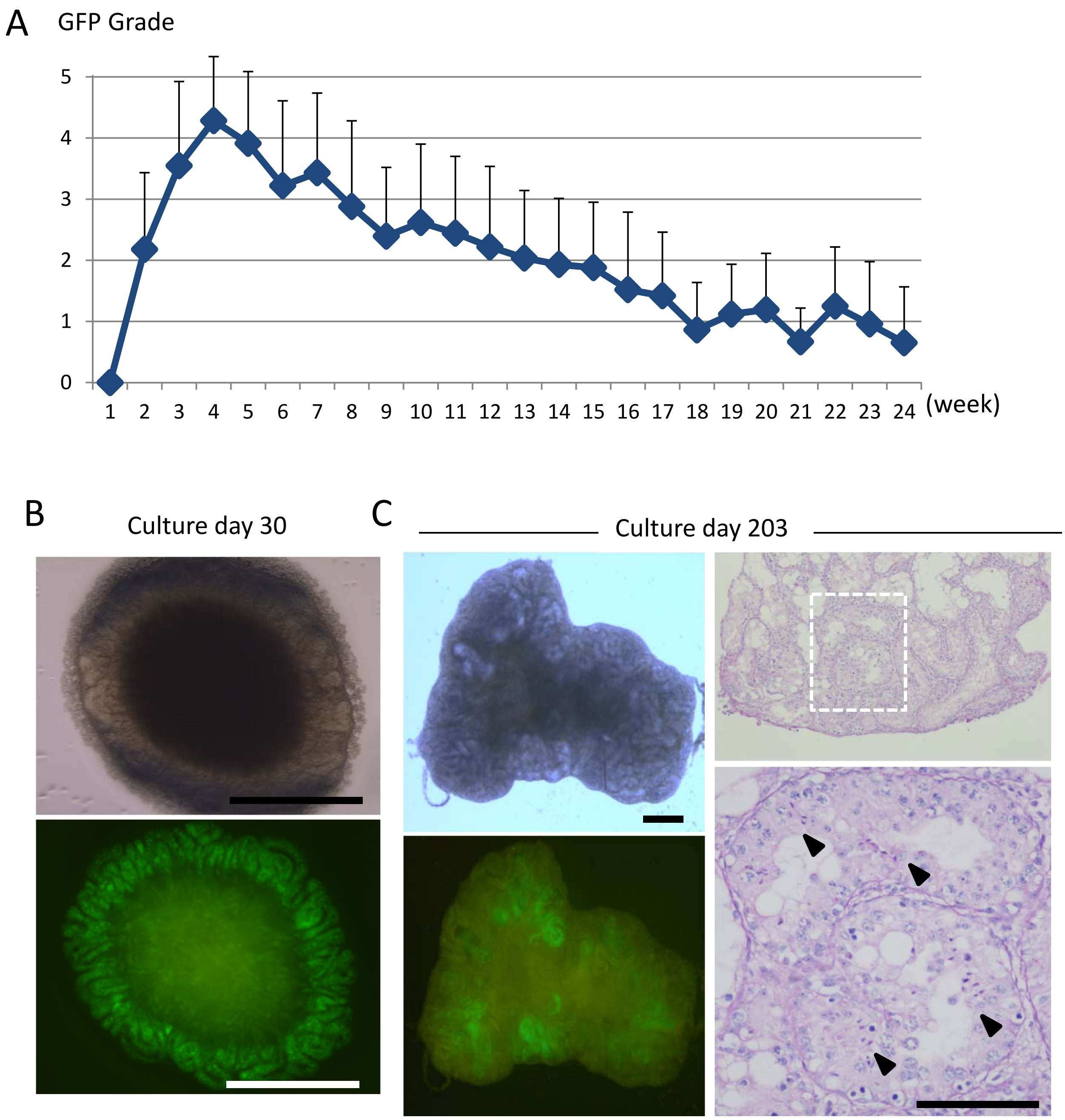

Fig. S3. Re-evaluation of conventional interphase method, agarose gel (AG) method. (*A*) Transition of the average GFP grade during long-term culture, performed in 16 experiments with 73 samples. (*B*) Testis tissue cultured on agarose gel for 30 days showed GFP-positive tubules in the peripheral area including the margin of the tissue mass. (*C*) In a few instances in which the GFP expression was maintained for a long period, the GFP was located not on the margin but in the submarginal region of the tissue. Histological examination also showed sperm-forming tubules located not at the surface but in the submarginal region. The dashed rectangular area is enlarged in the panel below. Arrowheads indicate elongated spermatids. Scale bars: 1 mm (*B*), 500 µm (*C*, left upper), 100 µm (*C*, right lower).

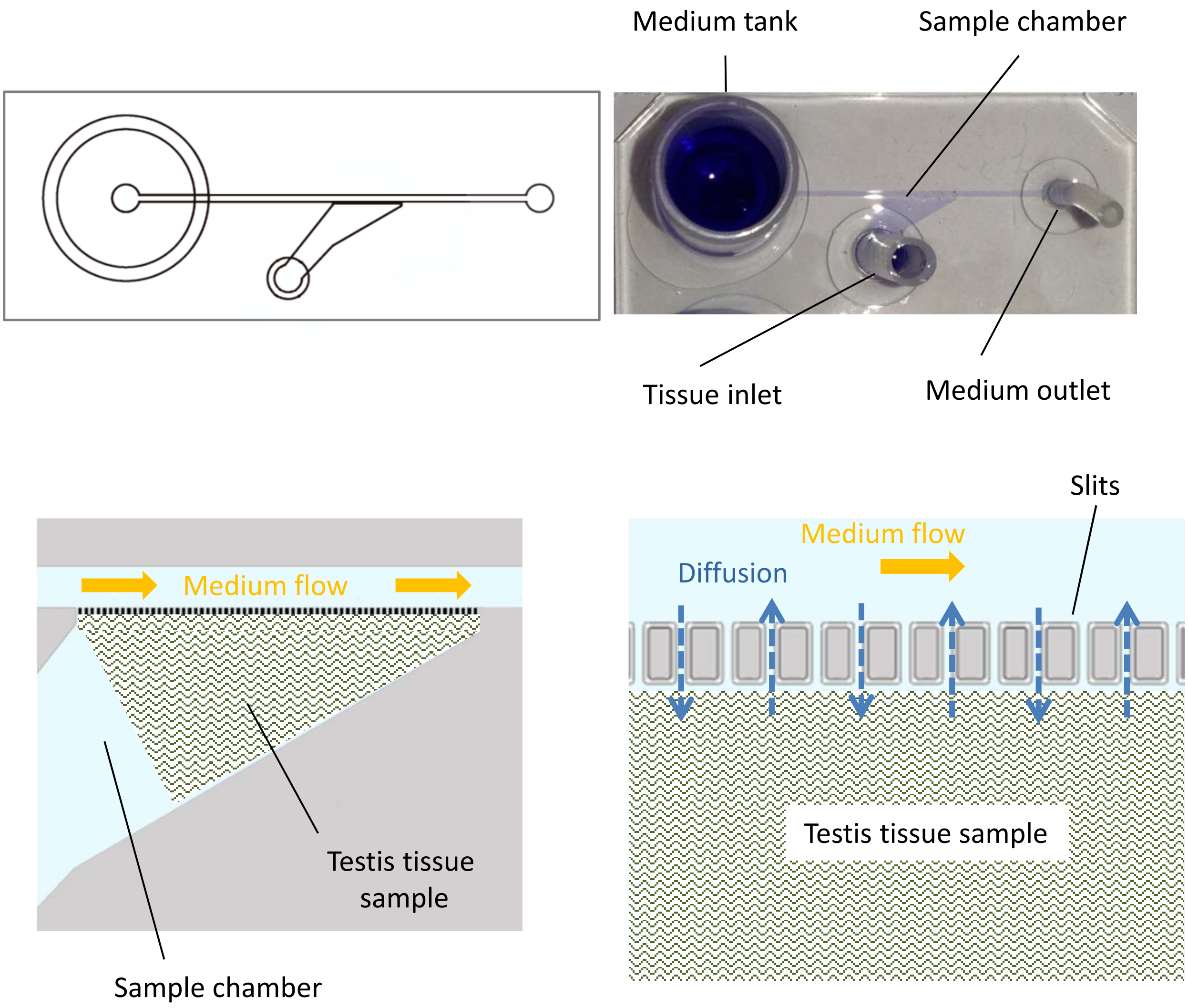

Fig. S4. Microfluidic device equipped with slit structure (Slit device). Medium flowed through the medium channel (500 µm in width and 80 µm in depth), which was separated from the tissue chamber by thin pillars. Each pillar was 50 µm wide, 100 µm long, and 80 µm in height. The slits between pillars were 25 µm wide.

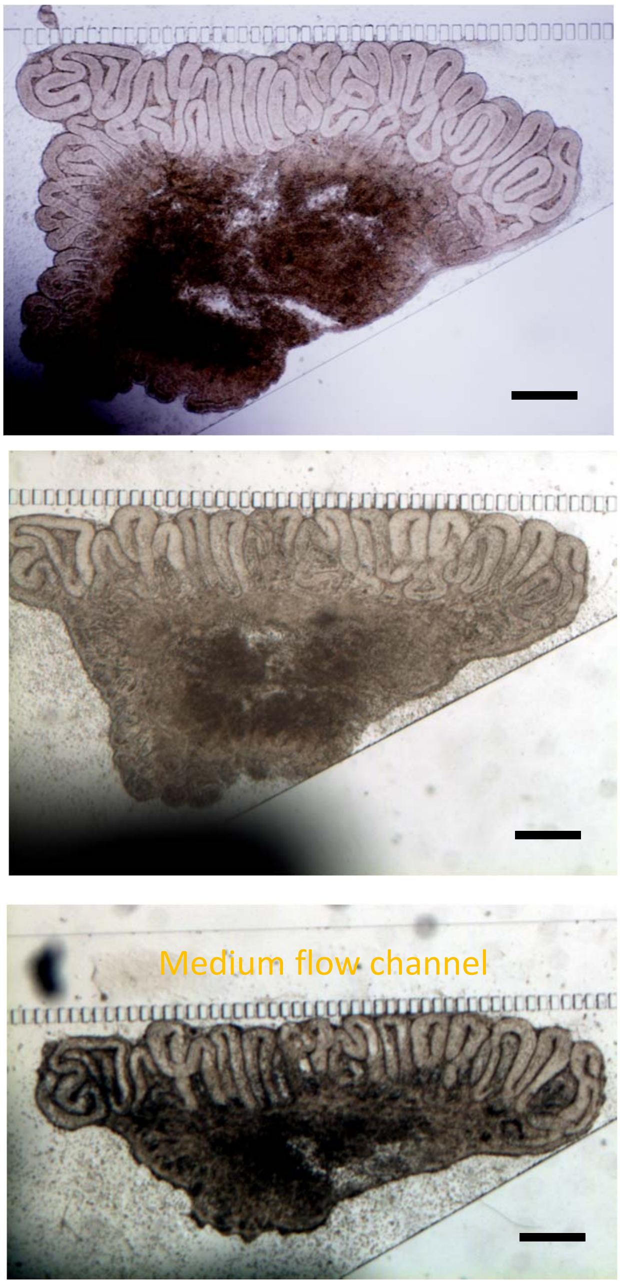

Day 13

Day 25

Day 44

Fig. S5. Molecular diffusion in testis tissue. Testis tissue was cultured in the slit device, and photos were taken on days 13, 25, and 44. The region of the tissue distant from the medium flow channel became necrotic due to a shortage of nutrients. Oxygen was supplied evenly through the ceiling made of PDMS. The distance of nutrient diffusion from the medium flow through the testis tissue to maintain it was about 400 µm at most in this experiment. Scale bar: 400 µm.

0.5 dpp

8.5 dpp

31.5 dpp

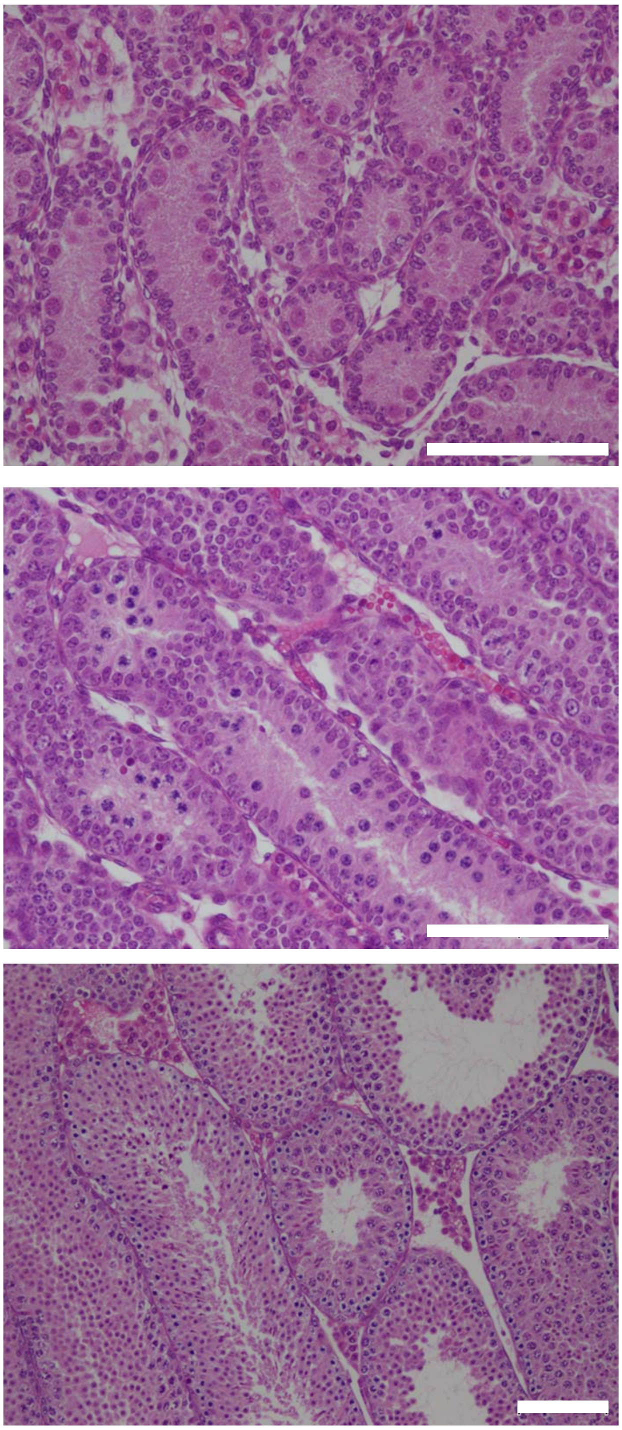

Fig. S6. Spermatogenic progression in the body. The neonatal testis used in the study, at 0.5 dpp, harbors gonocytes as germ cells. They became spermatogonia during the following days and meiotic entry was observed in 8.5 dpp testis. At 31.5 dpp, elongating spermatids could be observed and 4 layers of germ cells could be recognized inside the seminiferous tubule. Scale bar: 100 µm

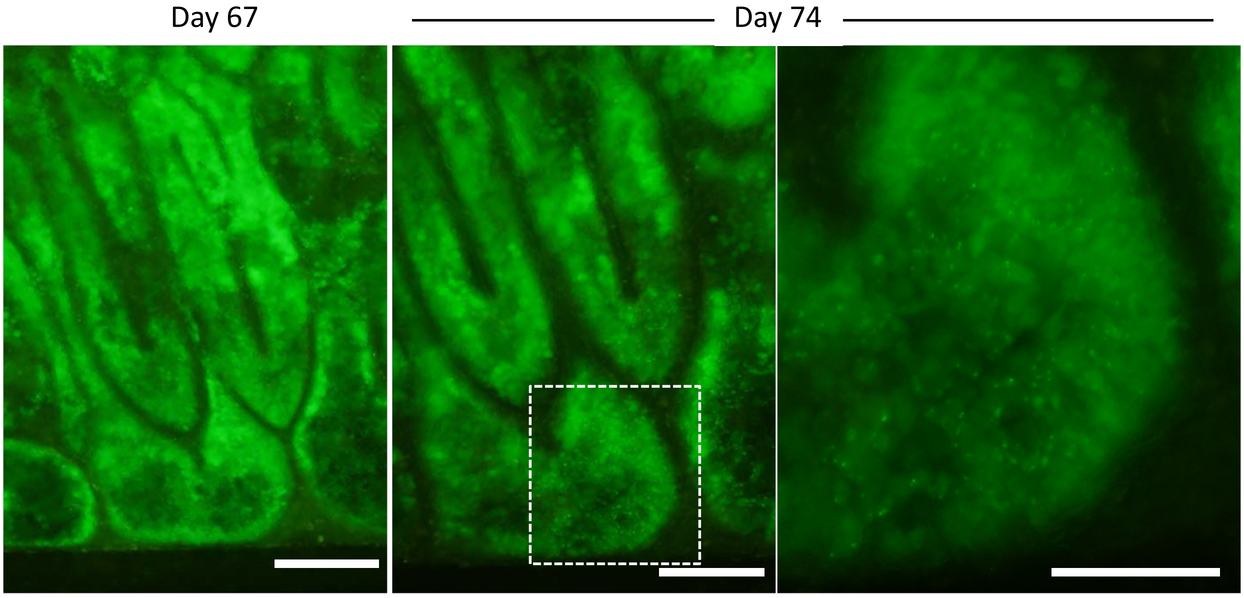

Fig. S7. Single-spot time-course observation. Neonatal, 1.5 dpp, mouse testis tissue was cultured in the microfluidic device. A single spot of cultured tissue was photographed on culture days 67 and 74. During these 7 days, haploid cells, judged by a GFP-positive acrosomal dot or cap, appeared in the dashed rectangular area. The right-most panel is a magnification of the area. Scale bars: 200 µm (left and center), 100 µm (right).

Table S1. Summary of micro-insemination experiment, culture duration 41 days

Micro-insemination	No. of oocytes inseminated	No. of oocytes developed into 2-cell embryos	No. of embryos transferred	No. of Implantations	No. of live offspring	Female /male
ROSI	29	29	29	15	9	6/3
ICSI	36	18	18	9	5	3/2

Table S2. Summary of micro-insemination experiment, culture duration 185 days

Micro-insemination	No. of oocytes inseminated	No. of oocytes developed into 2-cell embryos	No. of embryos transferred	No. of Implantations	No. of live offspring	Female /male
ROSI	99	89	61	21	6	3/3
ICSI	40	34	34	16	5	3/2
